# Walking speed is associated with postoperative pain catastrophizing in patients with lumbar spinal stenosis: a prospective observational study

**DOI:** 10.1186/s12891-022-06086-y

**Published:** 2022-12-20

**Authors:** Takashi Wada, Shinji Tanishima, Yuki Kitsuda, Mari Osaki, Hideki Nagashima, Hisashi Noma, Hiroshi Hagino

**Affiliations:** 1 Rehabilitation Division, Tottori University Hospital, 36-1 Nishi-Cho, Yonago, Tottori, 683-8504 Japan; 2 Division of Orthopedic Surgery, Department of Sensory and Motor Organs, School of Medicine, Faculty of Medicine, Tottori University, 36-1 Nishi-Cho, Yonago, Tottori, 683-8504 Japan; 3 Department of Data Science, The Institute of Statistical Mathematics, 10-3 Midori-Cho, Tachikawa, Tokyo, 190-8562 Japan; 4 School of Health Science, Faculty of Medicine, Tottori University, 86 Nishi-Cho, Yonago, Tottori, 683-8504 Japan

**Keywords:** Catastrophizing, Lumbar spinal stenosis, Lumbar surgery, Pain catastrophizing, Spine surgery, Walking speed

## Abstract

**Background:**

The purpose of this study was to investigate whether walking speed is associated with postoperative pain catastrophizing in patients with lumbar spinal stenosis.

**Methods:**

In this prospective observational study, consecutive patients with clinically and radiologically defined lumbar spinal stenosis underwent surgical treatment (decompression, or posterolateral or transforaminal lumbar interbody fusion) at Tottori University Hospital, between October 2015 and April 2018. The pain catastrophizing scale, walking speed, leg and back pain (numerical rating scale), and Japanese Orthopaedic Association score were evaluated preoperatively and at 3, 6, and 12 months postoperatively. Correlations between the pain catastrophizing scale and each variable were analyzed at each evaluation time point. The effect of walking speed on the pain catastrophizing scale was analyzed using mixed-effect models for repeated measurements.

**Results:**

Ninety-four patients were included at baseline, and 83, 88, and 82 patients were analyzed at 3, 6, and 12 months postoperatively, respectively. The pain catastrophizing scale was significantly correlated with walking speed, leg pain, back pain, and the Japanese Orthopaedic Association score at all evaluation time points. The pain catastrophizing scale was associated with walking speed at all evaluation time points.

**Conclusions:**

Our results suggest that changes in postoperative pain catastrophizing after lumbar spine surgery are associated with walking speed. Thus, walking speed is a necessary assessment for the management of pain catastrophizing and associated pain and disability in patients after lumbar spine surgery.

## Background

Pain catastrophizing refers to the tendency to view the experience of pain negatively [1]. The severity of pain catastrophizing in patients with lumbar spinal stenosis (LSS) is associated with low muscle mass and worsening pain and disability [2, 3]. In addition, the severity of pain catastrophizing in patients after lumbar spine surgery is associated with the severity of disability and a decreased quality of life [4]. Furthermore, it has been suggested that pain catastrophizing at 6 weeks postoperatively in patients after lumbar spine surgery predicts the intensity of lower back pain, pain interference, and disability at 6 months postoperatively [5].

Pain catastrophizing has been suggested as a relatively enduring factor [6]. In contrast, pain catastrophizing in patients undergoing surgery for LSS has been reported to be a dynamic entity that changes with pain and the Oswestry Disability Index [7]. Therefore, the management of pain catastrophizing after lumbar spine surgery is important for a good clinical outcome, and pain catastrophizing may be improved following surgery and rehabilitation.

Pain catastrophizing is associated with objective gait parameters. It has been reported that patients with LSS who have severe pain catastrophizing have a lower walking speed than those with mild pain catastrophizing [8]. Furthermore, pain catastrophizing is associated with excessive muscle activity of the erector spinae muscles during walking in patients with chronic lower back pain [9]. Walking speed is an easily available assessment and is known as the sixth vital sign that can be used as an indicator to predict future events [10]. The evaluation of walking speed is also recommended for patients with LSS as an indicator of improvement in postoperative mobility [11, 12]. However, previous studies have only investigated the relationship between pain catastrophizing, pain, and disability, and they have not longitudinally investigated the relationship between pain catastrophizing and changes in objective motor assessment after surgery.

In this study, we evaluated changes in pain catastrophizing and walking speed from the preoperative to postoperative periods in patients with LSS and aimed to investigate whether walking speed is related to postoperative changes in pain catastrophizing.

## Methods

### Participants

In this prospective observational study, consecutive patients with clinically and radiologically defined LSS underwent surgical treatment (decompression with/without posterolateral or transforaminal lumbar interbody fusion) between October 2015 and April 2018. The exclusion criteria were: (1) medical conditions that the investigator judged to affect the surgery or that might result in an abnormal postoperative course, such as stroke, neuromuscular disease, cancer, high risk of infection, or cardiovascular disease; (2) dementia or inability to answer the questionnaire independently; (3) previous lumbar spine surgery; and (4) inability to walk. Participant inclusion/exclusion criteria were similar to those of previous studies [13, 14]. Written informed consent was obtained from all participants. This study was approved by the Ethics Committee of the Tottori University Faculty of Medicine on August 31, 2015 (No. 1508B013).

### Demographic and clinical information

The age, sex, height, weight, body mass index (BMI), current smoking habits, employment status, symptom duration, comorbidities (hypertension, dyslipidemia, and diabetes), and type and level of surgery were collected from medical records. Leg numbness was assessed using the visual analogue scale [15]. Lower extremity muscle strength was assessed using manual muscle testing (MMT) [16]. Tibialis anterior, extensor hallucis longus, extensor digitorum longus, flexor hallucis longus, flexor digitorum longus, and gastrocnemius were targeted. Participants with an MMT grade of less than 5 (normal) were judged to have muscle weakness. Walking distance was used to assess intermittent claudication. Participants were measured for walking distance by walking a 90-m path until they could no longer walk due to pain or numbness. The maximum distance for this measurement was 500 m.

### Pain catastrophizing

Pain catastrophizing was assessed using the pain catastrophizing scale (PCS) [17]. The Japanese version of the PCS used in this study has also been shown to be reliable and valid [18]. Participants responded to 13 questions on a scale from 0 ("not at all") to 4 ("always"). The final score ranged from 0 to 52. The higher the PCS score, the more severe the catastrophic thoughts of pain. In this study, the PCS overall score and subscale scores for helplessness, magnification, and rumination were used.

### Walking speed

Walking speed was evaluated using a 10-m walking test. Walking speed was calculated from the time required to walk 10 m at a normal speed. Normal walking speed is a highly reliable metric [19].

### Pain and clinical outcomes

The intensity of leg and back pain due to LSS was assessed using the numerical rating scale (NRS) [20]. Clinical outcomes were assessed using the Japanese Orthopaedic Association (JOA) score for lower back pain [21]. The JOA score was calculated with a range of 0–29 points, with lower scores indicating a more severe disease.

### Data collection schedule

During the preoperative evaluation, which took place between admission and surgery, demographic data were collected, and the aforementioned PCS and NRS questionnaires and walking test were implemented. The results of the preoperative evaluation were used as the baseline data. The participants underwent general rehabilitation, including aerobic exercise, stretching, and strength training, for 2 weeks after surgery. The postoperative evaluation was performed at 3, 6, and 12 months postoperatively using the same aforementioned questionnaire and functional tests. The data collection schedule of this study was the same as that of a previous study [14].

### Statistical analysis

Data are presented as mean and standard deviation or median and interquartile range. Paired *t*-tests were used to compare the baseline and 12-month postoperative outcomes. In addition, Pearson correlation coefficients were used to assess the association between PCS and each variable at each evaluation time point and the association between the change in PCS and the change in each variable from baseline to 12 months postoperatively. In addition, the mixed-effect model for repeated measurements [22] was used to evaluate the association between PCS and walking speed at three time points during the follow-up period (3, 6, and 12 months postoperatively), adjusting for potential biases caused by missing data. In the multivariate analysis model, we modeled the following potential confounding factors as explanatory variables: age, sex, BMI, symptom duration, surgical treatment (decompression: 0, decompression with fusion: 1), NRS for leg and back pains, and preoperative PCS. We modeled the correlation matrix of outcome variables as the unstructured structure. All data were analyzed using SPSS version 24 for Windows (IBM Corporation, Armonk, NY, USA) and SAS version 9.4 (SAS Institute, Inc., Cary, NC). All *P*-values were two-sided, and the significance level was set at 0.05.

## Results

**Fig. 1:**
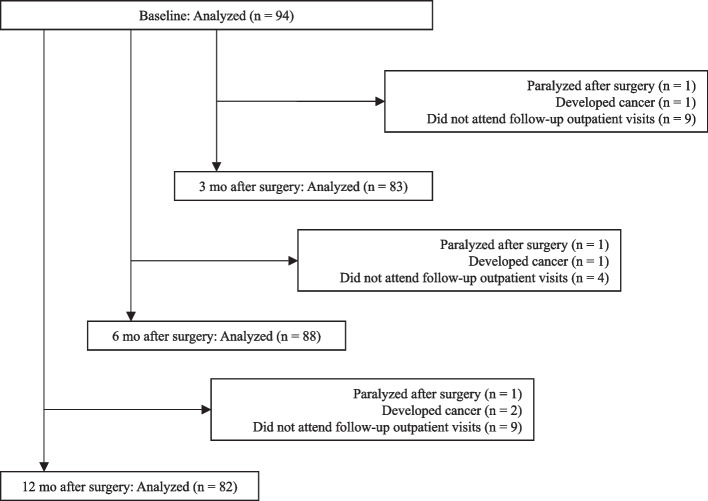
Study flowchart

Ninety-four patients were selected for inclusion at baseline, and 83, 88, and 82 patients were analyzed at 3, 6, and 12 months postoperatively, respectively (Fig. 1). Table 1 shows the demographic and clinical information at baseline. The mean postoperative rehabilitation time was 145.1 ± 66.8 min.

**Table 1:** Demographic and clinical information at baseline

	Baseline (*n* = 94)
Age (y)	69.6 ± 8.8
Female (%)	48.9
Height (cm)	157.9 ± 9.3
Weight (kg)	61.3 ± 11.6
BMI (kg/m^2^)	24.5 ± 3.1
Current smoking habit (%)	9.6
Employed (%)	35.1
Symptom duration (mo)	13.0 (6.0–46.5)
Hypertension (%)	48.9
Dyslipidemia (%)	22.3
Diabetes (%)	13.8
VAS score for leg numbness (mm)	70.0 (50.0–80.0)
Muscle weakness	
Tibialis anterior (n, %)	18 (19.1)
Extensor hallucis longus (n, %)	23 (24.5)
Extensor digitorum longus (n, %)	13 (13.8)
Flexor hallucis longus (n, %)	6 (6.4)
Flexor digitorum longus (n, %)	6 (6.4)
Gastrocnemius (n, %)	3 (3.2)
Walking distance (m)	200.0 (80.0–500.0)
Surgical treatment	
Decompression (n)	41
1 level (n, %)	27 (65.8)
2 levels (n, %)	10 (24.4)
3 levels (n, %)	4 (9.8)
Decompression with fusion (n)	53
1 level (n, %)	28 (52.8)
2 levels (n, %)	24 (45.3)
4 levels (n, %)	1 (1.9)

Data are presented as mean ± standard deviation or median (interquartile range)

*BMI* Body mass index, *VAS* Visual analogue scale

Table 2 shows the comparisons of pain catastrophizing, walking speed, pain, and clinical outcomes between baseline and 12 months postoperatively. All variables showed significant improvement 12 months after surgery. Table 3 shows the correlation between PCS and each variable at each evaluation time point. PCS was significantly correlated with walking speed, leg pain, back pain, and JOA score at all evaluation time points. The change in PCS from baseline to 12 months postoperatively was significantly correlated with changes in walking speed, leg pain, back pain, and JOA score.

**Table 2:** Changes in pain catastrophizing, walking speed, pain intensity, and clinical outcomes before and after surgery

	Baseline (*n* = 94)	12 months after surgery (*n* = 82)	*P*-value
PCS			
Helplessness	10.6 ± 4.8	5.2 ± 5.0	< 0.001
Magnification	6.2 ± 2.9	3.6 ± 3.2	< 0.001
Rumination	15.9 ± 3.8	8.9 ± 6.7	< 0.001
Total	32.7 ± 10.2	17.7 ± 14.2	< 0.001
Walking speed (m/s)	0.97 ± 0.27	1.16 ± 0.26	< 0.001
NRS score for leg pain	5.7 ± 2.5	2.7 ± 2.7	< 0.001
NRS score for back pain	5.3 ± 2.5	2.6 ± 2.5	< 0.001
JOA score	15.4 ± 4.1	21.0 ± 5.2	< 0.001

Data are presented as mean ± standard deviation

*PCS* Pain Catastrophizing Scale, *NRS* Numerical rating scale, *JOA* Japanese Orthopaedic Association

**Table 3:** Correlation between pain catastrophizing and walking speed, pain intensity, and clinical outcomes

Time point	Outcome	Preoperative PCS	PCS at 3 months after surgery	PCS at 6 months after surgery	PCS at 12 months after surgery	Change in PCS at 12 months
Preoperative (*n* = 94)	Walking speed	-0.20*				
	NRS for leg pain	0.25*				
	NRS for back pain	0.27*				
	JOA score	-0.26*				
3 months after surgery (*n* = 83)	Walking speed		-0.31**			
	NRS for leg pain		0.43**			
	NRS for back pain		0.42**			
	JOA score		-0.59**			
6 months after surgery (*n* = 88)	Walking speed			-0.33**		
	NRS for leg pain			0.73**		
	NRS for back pain			0.64**		
	JOA score			-0.63**		
12 months after surgery (*n* = 82)	Walking speed				-0.40**	
	NRS for leg pain				0.69**	
	NRS for back pain				0.66**	
	JOA score				-0.74**	
Change in walking speed at 12 months						-0.25*
Change in NRS for leg pain at 12 months						0.40**
Change in NRS for back pain at 12 months						0.53**
Change in JOA score at 12 months						-0.45**

Values indicate R (correlation coefficient)

*PCS* Pain Catastrophizing Scale, *NRS* Numerical rating scale, *JOA* Japanese Orthopaedic Association

^*^P < 0.05; ^**^P < 0.01

Table 4 shows the results of the multivariate analysis for the association between PCS and walking speed, as well as the adjusted mean change rate of PCS per 1 m/sec at 3, 6, and 12 months by the mixed-effect model for repeated measurements. PCS was significantly associated with the walking speed at all evaluated time points.

**Table 4:** Association between postoperative pain catastrophizing and walking speed

Time	Estimate	95% CI	*P*-value
3 months	-17.45	-29.58 to -5.33	0.005
6 months	-17.26	-28.28 to -6.23	0.002
12 months	-19.65	-30.60 to -8.71	< 0.001

Adjusted mean change rate estimate of pain catastrophizing scale per 1 m/sec by mixed-effect model for repeated measurements

*CI* Confidence interval

## Discussion

This study showed that pain catastrophizing significantly improved postoperatively. Walking speed showed a significant negative correlation with PCS before and at 3, 6, and 12 months after surgery. Furthermore, the PCS at each postoperative assessment time point was associated with walking speed.

Pain catastrophizing is controversial, with reports of "relatively enduring" characteristics that do not change before and after surgery [6, 23] and reports of "dynamic" characteristics that do change [7]. According to the present study, pain catastrophizing was significantly reduced after surgery and rehabilitation for LSS. This result may be due to the high preoperative PCS values of the participants in our study, which made them more susceptible to changes over time due to surgery and rehabilitation.

PCS was correlated with walking speed at each assessment time point. Furthermore, postoperative PCS was affected by walking speed, which is an objective measure of motor function. This is the novelty of our study, which differs from previous studies that have investigated the relationship between PCS and the intensity of pain and disability using a questionnaire [7]. Walking speed in patients with lumbar spine disease improves with surgery and rehabilitation, similar to pain reduction [24–26]. Therefore, the excessive fear of pain postoperatively in patients with LSS may have decreased as their walking speed improved with surgery and rehabilitation. It is also possible that increased physical activity with improved walking speed contributed to the change in pain catastrophizing in patients after surgery for LSS. Spine surgery for LSS has been reported to improve postoperative physical activity levels, even in patients with low preoperative levels of physical activity [27]. A study of patients with chronic low back pain reported higher PCS in patients with low physical activity compared to those with high physical activity [28]. Therefore, pain catastrophizing may have improved as a result of improved walking speed due to surgery and rehabilitation, which contributed to increased physical activity. The results of this study suggest that the postoperative management of pain catastrophizing in patients with LSS may require improving motor function through continuous rehabilitation and acquisition of exercise habits in addition to pain management. Although pain catastrophizing in postoperative patients with LSS has received attention for its relationship with pain intensity and degree of disability, walking speed may need to be assessed over time.

This study had several limitations. First, the sample size was small, and there was a potential dropout bias since there were 11 dropouts at 3 months, postoperatively; 6 at 6 months, postoperatively; and 12 at 12 months, postoperatively. This may have affected the results. Second, we were unable to measure the amount of physical activity. Questionnaires and pedometers should be used to assess the amount of physical activity in future studies. Third, there was a lack of detailed spinal stenosis assessment of LSS. Fourth, intermittent claudication was assessed only by walking distance. A more detailed evaluation using the Zurich claudication questionnaire is needed in future studies.

## Conclusions

We investigated whether changes in pain catastrophizing after surgery were associated with walking speed in patients with LSS. Our results suggest that changes in postoperative pain catastrophizing are associated with walking speed. Thus, walking speed assessment may be necessary for the management of postoperative pain catastrophizing.

## Data Availability

The datasets generated during and/or analyzed during the current study are available from the corresponding author on reasonable request.

## Abbreviations

BMI: Body mass index
JOA: Japanese Orthopaedic Association
LSS: Lumbar spinal stenosis
NRS: Numerical rating scale
PCS: Pain catastrophizing scale
